# Tenotomy or Tenodesis for Tendinopathy of the Long Head of the Biceps Brachii: An Updated Systematic Review and Meta-analysis

**DOI:** 10.1016/j.asmr.2021.02.010

**Published:** 2021-07-03

**Authors:** Bauke Kooistra, Navin Gurnani, Alexander Weening, Derek van Deurzen, Michel van den Bekerom

**Affiliations:** a Department of Orthopaedic Surgery, Medische Kliniek Velsen, Velsen-Noord, the Netherlands; b Department of Orthopaedic Surgery, Onze Lieve Vrouwe Gasthuis, Amsterdam, the Netherlands; c Department of Orthopaedic Surgery, Vrije Universiteit Medical Centre, Amsterdam, the Netherlands

## Abstract

**Purpose:**

The purpose of this meta-analysis was to provide an up-to-date comparison of clinical outcomes of tenotomy and tenodesis in the surgical treatment of long head of the biceps brachii (LHB) tendinopathy.

**Methods:**

A literature search was conducted in EMBASE, Pubmed/Medline and the Cochrane database from January 2000 to May 2020. All studies comparing clinical outcomes between LHB tenotomy and tenodesis were included. Quality was assessed using the Coleman score.

**Results:**

We included 25 studies (8 randomized studies) comprising 2,191 patients undergoing LHB tenotomy or tenodesis, with or without concomitant shoulder procedures (mainly rotator cuff repairs). The Coleman score ranged from 29 to 97 for all studies. When comparing tenodesis and tenotomy in randomized studies, no clinically relevant differences were found in the Constant score (mean difference, 0.9 points), the American Shoulder and Elbow Society Score (mean difference, 1.1 points), shoulder pain (mean difference in visual analogue scale, -0.3 points), elbow flexion strength loss (mean difference, 0%), or forearm supination strength (mean difference, 3%). A Popeye deformity (odds ratio, 0.32) was less commonly seen in patients treated with tenodesis (9% vs 23%).

**Conclusion:**

In our meta-analysis, a Popeye deformity was more frequently observed in patients treated with tenotomy. Based on a substantial number of studies, there is no evidence-based benefit of LHB tenodesis over tenotomy in terms of shoulder function, shoulder pain or biceps-related strength. It is unclear whether LHB tenodesis is of benefit in specific patient groups such as younger individuals.

**Level of evidence:**

Level III, systematic review of level III or higher studies.

Tendinopathy of the long head of the biceps brachii (LHB) is a highly prevalent pathology in patients with anterior and deep shoulder pain.^1^, ^2^, ^3^ Additionally, it is associated with rotator cuff tears and superior labrum anterior-to-posterior lesions, possibly because of load alterations within the LHB tendon due to muscle-tendon imbalance in the shoulder joint and because of the close anatomic relationship of these structures.^4^ The surgical treatment of LHB tendinopathy, whether or not associated with rotator cuff tears or rotator cuff tendinopathy, consists of arthroscopic debridement combined with either tenotomy or tenodesis of the LHB. Tenodesis has been favored by some because of potentially greater elbow flexion and forearm supination strength, less cramping pain and less risk of Popeye deformity.Yet disadvantages (longer surgical time, longer rehabilitation, higher costs, cramping pain, and persistent pain in the bicipital groove) have also been reported.^2^ However, a meta-analysis performed in 2015 that included 650 patients from 9 studies, of which only 1 was a randomized controlled trial (RCT), did not show clinically relevant differences in Constant Score, elbow flexion or forearm supination strength. After tenodesis, patients did have lower probabilities of Popeye deformity (odds ratio [OR] 0.17) and cramping pain (OR 0.38).^5^ Since then, several new comparative studies, including 7 RCTs, of LHB tenotomy and tenodesis have been published.

The purpose of this meta-analysis was to provide an up-to-date comparison of clinical outcomes of tenotomy and tenodesis in the surgical treatment of LHB tendinopathy. The primary hypothesis was that LHB tenotomy and tenodesis would show no difference in shoulder function, shoulder pain or biceps-related strength. Moreover, we hypothesized that after LHB tenodesis, patients would be less likely to experience cramping bicipital pain and Popeye deformities.

## Materials and Methods

### Search Strategy

**Fig 1:**
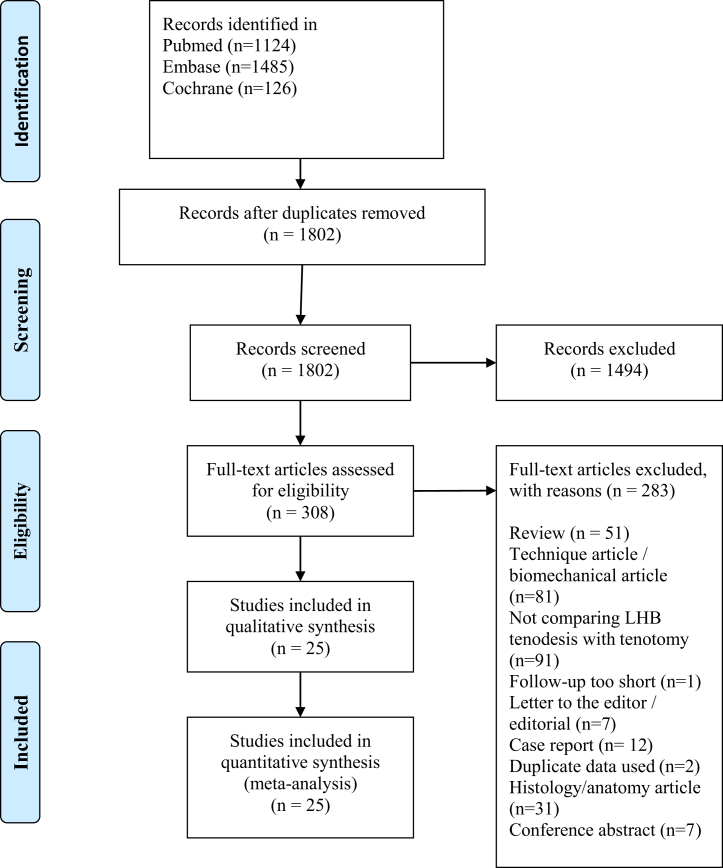
Flow diagram of study selection.

Our original study protocol was a priori registered at the International Prospective Register of Systematic Reviews (PROSPERO) (http://www.crd.york.ac.uk/), number CRD42018087257. This systematic review was conducted according to the PRISMA guidelines.^6^ EMBASE, MEDLINE and Cochrane databases were searched for studies comparing LBH tenodesis with tenotomy, published from inception until May 24, 2020. The search strategy can be found in the Appendix. Studies that described concomitant shoulder procedures, such as rotator cuff repair or labral repair, were included. Eligible studies had a minimum of 20 patients and a minimum follow-up of 12 months. The diagnosis of biceps tendinopathy had to be based on patient history, physical examination, ultrasound, MRI scan, or arthroscopic findings. Studies in languages other than English, Dutch or French were excluded (Fig 1). Two reviewers (NG and MB) searched the titles and abstracts for relevant studies. The full-text papers were examined by two authors (BWK and NG), and consensus was reached by discussion with the coauthors. Additionally, bibliographies of all obtained full-text articles were hand-searched for potential additional relevant studies.

### Outcome Measures

For conciseness, we chose to report only on outcomes that had been reported in a minimum of 3 studies. These were:

1. Constant score, with a minimal clinically important difference of 10 to 17 points^7^, ^8^, ^9^
2. American Shoulder and Elbow Society Score^10^
3. Elbow strength index (ESI),^11^ representing the ratio of the strength (measured in kilograms, Newtons, Newton-meters, or pounds) of elbow flexion on the affected side and the contralateral side during a single measurement
4. Forearm supination strength index (FSSI),^11^ representing the ratio of the strength (measured in kilograms, Newtons, Newton-meters, or pounds) of forearm supination on the affected side and the contralateral side during a single measurement, expressed in Nm
5. Presence of a Popeye deformity
6. Presence of cramping pain in the biceps muscle
7. Shoulder pain, expressed as the Visual Analogue Scale (VAS), ranging from 0 to 10.^12^

### Data Collection

Two reviewers (BK and NG) extracted the data from the included papers. The investigations and their credentials were assessed by BK. Articles were not blinded for author, affiliation or source. If standard deviation was not mentioned, it was calculated based on the confidence interval.^13^

### Assessment of Risk of Bias

The Coleman methodology score was used to determine the methodologic quality of included studies, with total scores ranging from 0 (worst score) to 100 (best score). The Coleman scoring system has been validated in various research facilities and is reproducible and accurate.^14^^,^^15^ The studies were scored by 3 reviewers (BK, NG and AW). Discrepancies were resolved by consensus.

### Statistical Analysis

Study outcomes of RCTs were pooled when the outcome was reported by 3 or more studies. We used random effects models because we identified clinical heterogeneity among the included studies. ORs were reported for dichotomous outcomes, and mean differences (MDs) were reported for continuous outcomes measurements, along with corresponding 95% confidence intervals (95% CI) and 95% prediction intervals (95% PI). Forest plots were generated for each outcome index. Heterogeneity was assessed using the ꭓ^2^ test.

We reported only outcomes of nonrandomized studies if certain outcome parameters were used in 3 or more studies. We did not pool outcomes of nonrandomized studies, but we reported the outcomes as ranges. Also, we created forest plots without pooled effect sizes, but we did calculate heterogeneity, expressed as the I^2^ statistic. We explored heterogeneity using subgroup analysis by minimum length of follow-up (<2 years vs ≥2 years), mean age (<60 years vs ≥60 years), rate of concurrent cuff repairs (<10% vs ≥10% of patients), rate of cointerventions, including cuff repair (<10% vs ≥10% of patients), and type of tenodesis (subpectoral vs suprapectoral and intracuff). We chose the cut-off of 10% of concurrent cuff repairs and other cointerventions because we felt that such a low rate of cointerventions would not substantially influence the overall effect of the type of biceps treatment and because only very few studies would actually contain only patients without any cointerventions.

Statistical significance was defined as *P* ≤ 0.05. To explore the effect of heterogeneity, sensitivity analyses were performed. Review Manager 5.2 (The Nordic Cochrane Centre, Copenhagen, Denmark; The Cochrane Collaboration) and R Project for Statistical Computing software (RStudio, version 1.2.1335; R Foundation for Statistical Computing, Vienna, Austria) were used for meta-analysis.

## Results

### Included Studies

The characteristics of the studies are summarized in Table 1. Twenty-five studies reporting on 2,191 participants were included in this meta-analysis.^16^, ^17^, ^18^, ^19^, ^20^, ^21^, ^22^, ^23^, ^24^, ^25^, ^26^, ^27^, ^28^, ^29^, ^30^, ^31^, ^32^, ^33^, ^34^, ^35^, ^36^, ^37^, ^38^, ^39^, ^40^, ^41^ Of these, 1,003 patients were treated with tenodesis (46%), and 1,188 were treated with tenotomy (54%). There were 8 level I studies, 5 level II studies and 12 level III studies. The majority of the participants were treated for biceps pathology with concomitant rotator cuff lesions. Three studies included only patients with isolated LHB tendinopathy. In total, patients treated by tenodesis or tenotomy had similar rates of concomitant shoulder procedures. For all patients, follow-up ranged from 1 to 10 years.

**Table 1:** Characteristics of the Included Studies

Author	Study type, LoE	Coleman	N	Outcomes	Included in previous review?^5^	Minimum FU (yr)	Mean age	Rate of patients with concurrent cuff repair (%)	Rate of patients with cointerventions (%)	Tenodesis type
Belay et al.	Randomized controlled triaI, I	66	34	VAS, ASES, SANE	No	2	56	56	56	Suprapectoral
Castricini et al.	Randomized controlled trail, I	91	55	Constant score, popeye deformity VAS, SF 36, ROM, elbow flexion strength, cramping pain	No	2	58	100	100	Suprapectoral
Hufeland et al.	Randomized controlled triaI, I	78	20	Constant score, flexion strength, and Popeye deformity	No	1	52	0	0	Suprapectoral
Lee et al.	Randomized controlled trial, I	94	128	ROM, VAS, ASES, Constant score, Popeye deformity	No	1	63	100	100	Suprapectoral
MacDonald et al.	Randomized controlled triaI, I	78	114	ASES, WORC, VAS, elbow flexion and supination strength (no comparison with contralateral side), Popeye	No	2	57	65	100	Subpectoral
Oh et al.	Randomized controlled trial, I	86	86	ASES, VAS, flexion strength, supination strength, Popeye deformity, cramping pain and bicipital pain	No	1	59	100	100	Suprapectoral
Van Deurzen et al.	Randomized controlled triaI, I	78	100	Constant sore, ESI, DASH, DOSS, EQ5D, VAS, external rotation, Popeye deformity	No	1	61	100	100	Intracuff
Zhang et al.	Randomized controlled trail, I	97	151	Surgical time, cost, pain (VAS), Popeye sign, flexion and supination strength and Constant score	yes	2	61	100	100	Suprapectoral
Aflatooni et al.	Retrospective cohort study, III	66	215	Satisfaction, cramping pain, and bicipital pain	No	1.8	61	55	65	Suprapectoral
Biz et al.	Prospective cohort study, II	33	252	Modified UCLA, VAS, SST, Popeye deformity, bicipital and cramping pain	No	1	57	100	100	Intracuff
Boileau et al.	Retrospective cohort study, III	85	72	Constant score, ROM, biceps related pain, radiologic changes, muscle cramps, Popeye deformity	yes	2	70	0	0	Suprapectoral
Cho et al.	Retrospective cohort study, III	76	83	Constant score, UCLA score and Popeye deformity, function, strength and acromiohumeral distance	yes	1.3	61	100	100	Intracuff
De Carli et al.	Prospective cohort study, II	66	65	Strength, Constant score, Popeye deformity	yes	1.6	58	100	100	Intracuff
Delle Rose et al.	Retrospective cohort study, III	71	104	Constant score, VAS and DASH and cramping pain, Popeye deformity	yes	2.4	48	0	0	Intracuff
Fang et al.	Retrospective cohort study, III	58	154	VAS, Constant score, ASES, DASH	No	1	63	100	100	Subpectoral
Friedman et al.	Retrospective cohort study, III	64	42	Popeye deformity, strength, ROM, VAS, DASH, ASES, cramping pain, and bicipital pain	No	1.6	49	62	91	Subpectoral
Godenèche et al.	Retrospective cohort study, III	71	134	Constant score, SST and SSV	No	10	56	100	100	Suprapectoral
Ikemoto et al.	Retrospective cohort study, III	57	77	UCLA, ROM and elbow flexion strength, Popeye deformity	No	2	58	100	100	Intracuff
Kerschbaum et al.	Prospective cohort study, II	55	57	Constant score, LHB score, Popeye deformity, bicipital and cramping pain	No	2	61	39	79	Suprapectoral
Koh et al.	Prospective cohort study, II	75	84	Popeye deformity, strength, Constant score, ASES	yes	2	65	100	100	Suprapectoral
Mardani et al.	Prospective Cohort study, II	53	44	Constant score, NRS, SST, cramping pain and Popeye deformity	No	2	55	100	100	Subpectoral
Meraner et al.	Retrospective cohort study, III	51	53	Constant score, flexion strength, cramping pain, and bicipital pain	No	2.2	58	100	100	Suprapectoral
Sentürk et al.	Retrospective cohort study, III	45	20	Strength, Constant and UCLA scores, Popeye deformity	yes	1	60	90	100	Suprapectoral
Shank et al.	Retrospective cohort study, III	58	67	Elbow flexion strength	yes	0.7	51	9	100	Suprapectoral
Wittstein et al.	Retrospective cohort study, III	72	35	Strength, SANE and MASES score, Popeye deformity	yes	2	57	51	51	Suprapectoral

ASES, American Shoulder and Elbow Score; Coleman, Coleman Score; DASH, Disabilities of the Arm, Shoulder and Hand; LHB, long head of the biceps brachii; LoE, level of evidence; MASES, Mid-Atlantic Shoulder & Elbow Society; NRS, numerical rating scale; ROM, range of motion; SANE, single assessment numeric evaluation; SST, simple shoulder score; SSV, subjective shoulder value; UCLA, University of California at Los Angeles score; VAS, Visual Analogue Score; WORC, Western Ontario Rotator Cuff index questionnaire.

### Quality Assessment

The Coleman score ranged from 29 to 97. The surgical procedure relating to the LHB tendon was described adequately (that is, in detail) in 14 studies; fairly (that is, mentioning only implants and approach) in 9 studies; and inadequately (that is, not mentioning anything) in 2. Fifteen studies reported the use of an independent outcome assessor. Six studies did not report patient recruitment adequately.

### Constant Score

**Fig 2:**
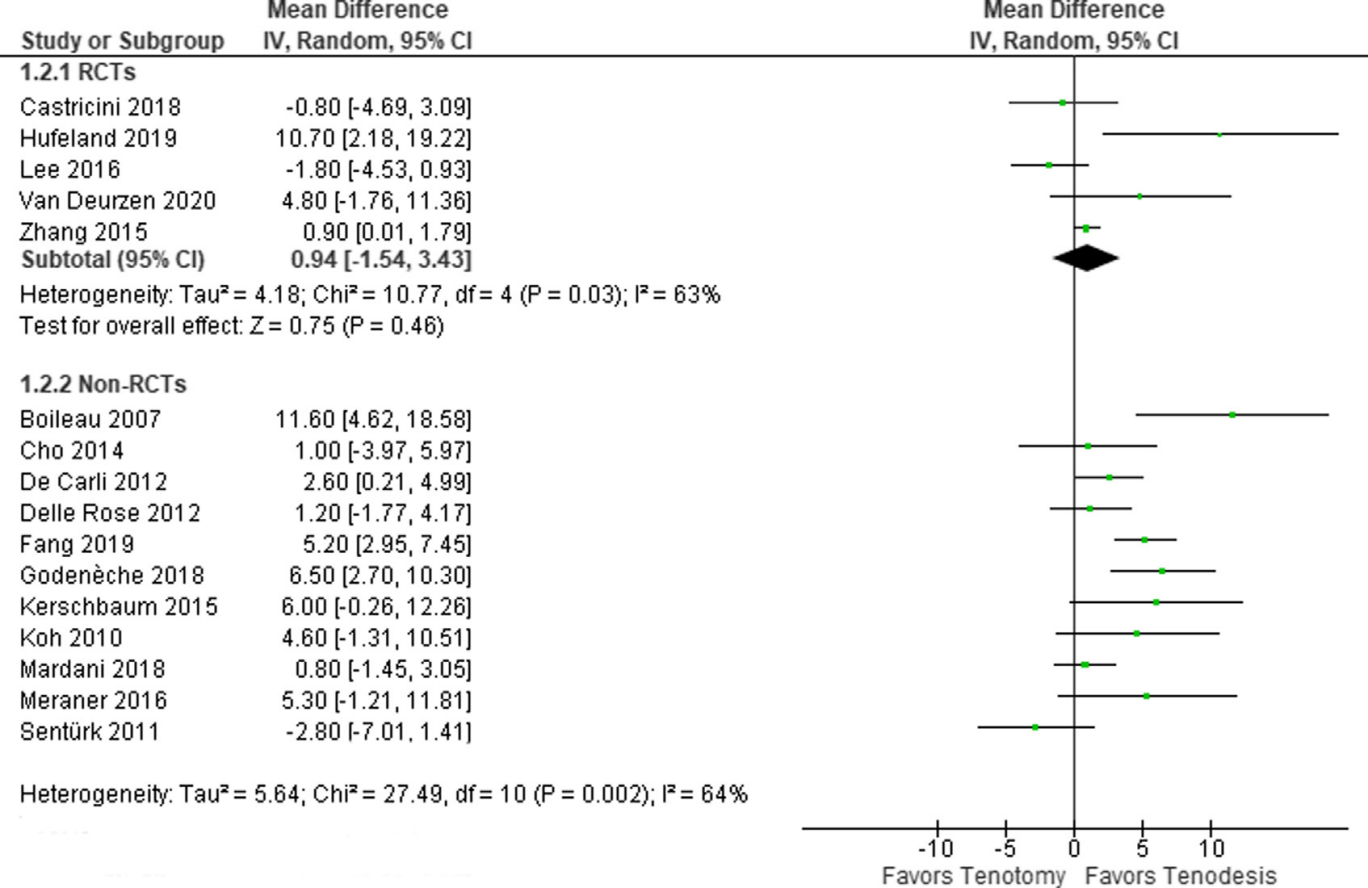
Forest plot of the Constant score.

The Constant score was reported in 16 studies including 1,370 patients. In a meta-analysis of 5 RCTs (434 patients), the Constant score was similar for both groups (MD, 0.9 points) (95% CI, -1.5 to 3.4 points; 95% PI, -6.7 to 8.6 points) (Fig 2). This difference is smaller than the minimal clinically important differences.

For the nonrandomized studies, the mean difference in the Constant score ranged from -2.8 to 11.6 points in favor of tenodesis (I^2^ = 64%). Subgroup analysis did not decrease heterogeneity.

### ASES Score

**Fig 3:**
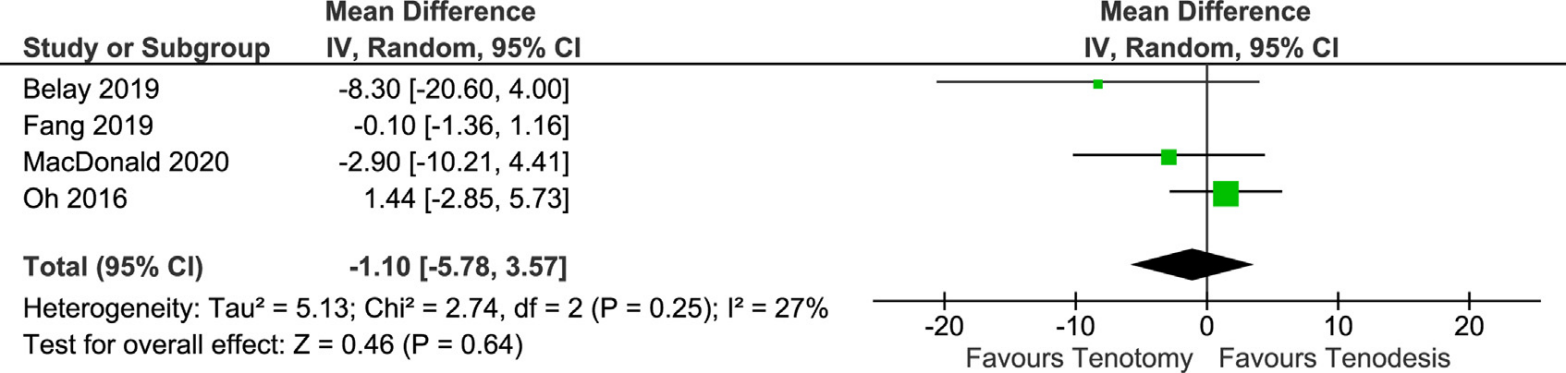
Forest plot of the American Shoulder & Elbow Surgeons score.

The American Shoulder and Elbow Surgeons (ASES) score was reported in 4 studies including 360 patients. In a meta-analysis of 3 RCTs (206 patients), the ASES score was similar for both groups (MD, -1.1 points; 95% CI, -5.8 to 3.6 points; 95% PI, -42.9 to 40.7 points) (Fig 3).

### Shoulder Pain

**Fig 4:**
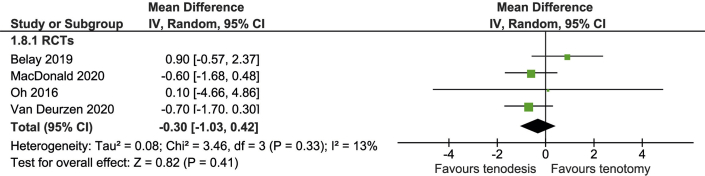
Forest plot of shoulder pain (Visual Analogue Scale).

Shoulder pain was reported in 5 studies including 454 patients. In a meta-analysis of 4 RCTs (300 patients), VAS for shoulder pain was similar for both groups (MD, -0.3 points (95% CI, -1.0 to 0.4 points; 95% PI, -2.3 to 1.7 points) (Fig 4). This is not clinically significant.

### Popeye Deformity

**Fig 5:**
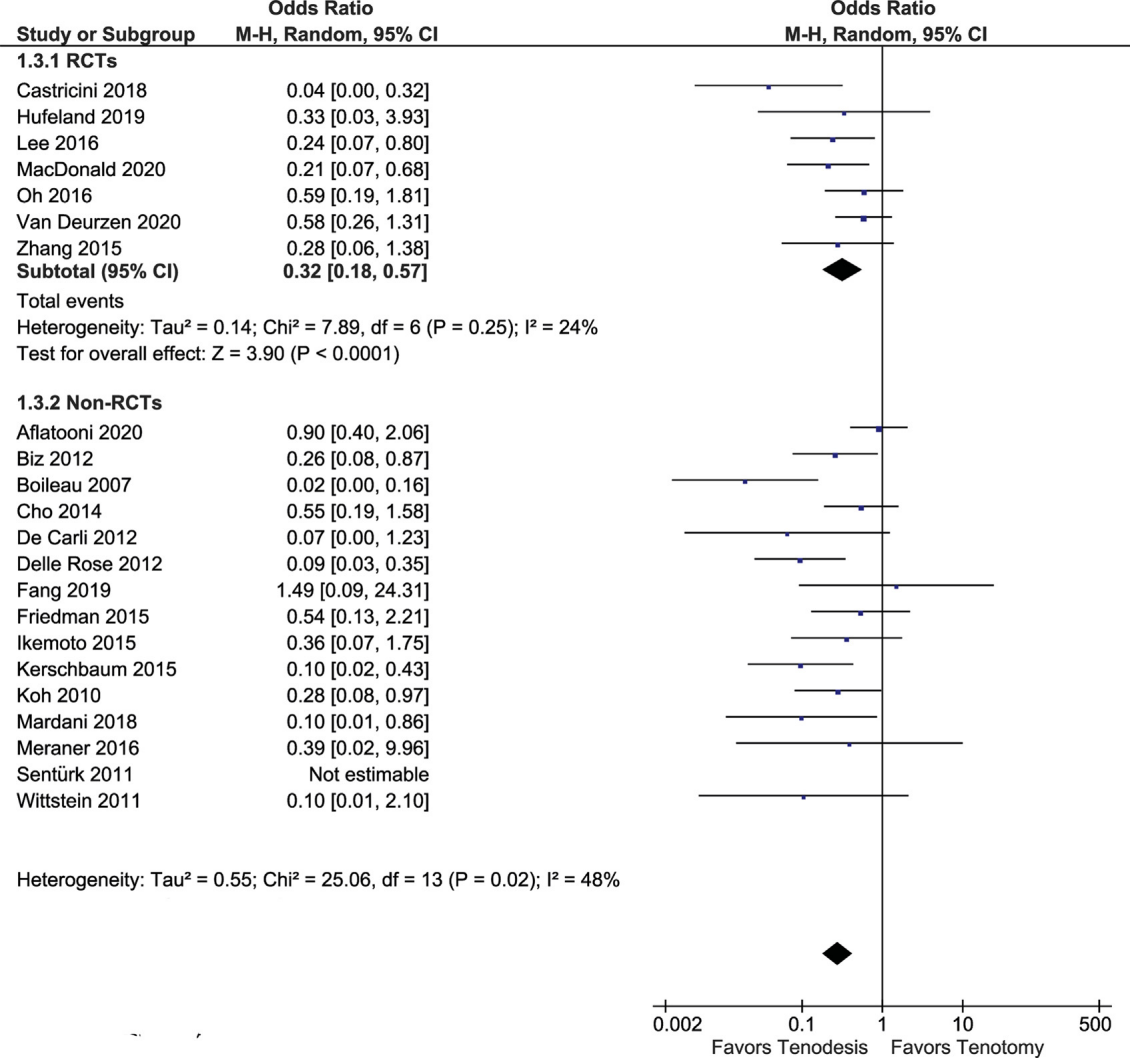
Forest plot of the presence of a Popeye deformity.

Popeye deformity was reported in 22 studies including 1,370 patients. In a meta-analysis of 7 RCTs (627 patients), Popeye deformity occurred more commonly in patients after tenotomy (OR, 0.32 points (95% CI, 0.18-0.57 points; 95% PI, 0.10-1.08) (Fig 5). In the population included in our meta-analysis, 23% of patients developed a Popeye deformity after tenotomy as compared to 9% after tenodesis. For the nonrandomized studies, the OR ranged from 0.02 to 1.49 (I^2^ = 48%). Including only studies with at least 2 years of follow-up decreased heterogeneity substantially (I^2^ = 2%).

### ESI

**Fig 6:**
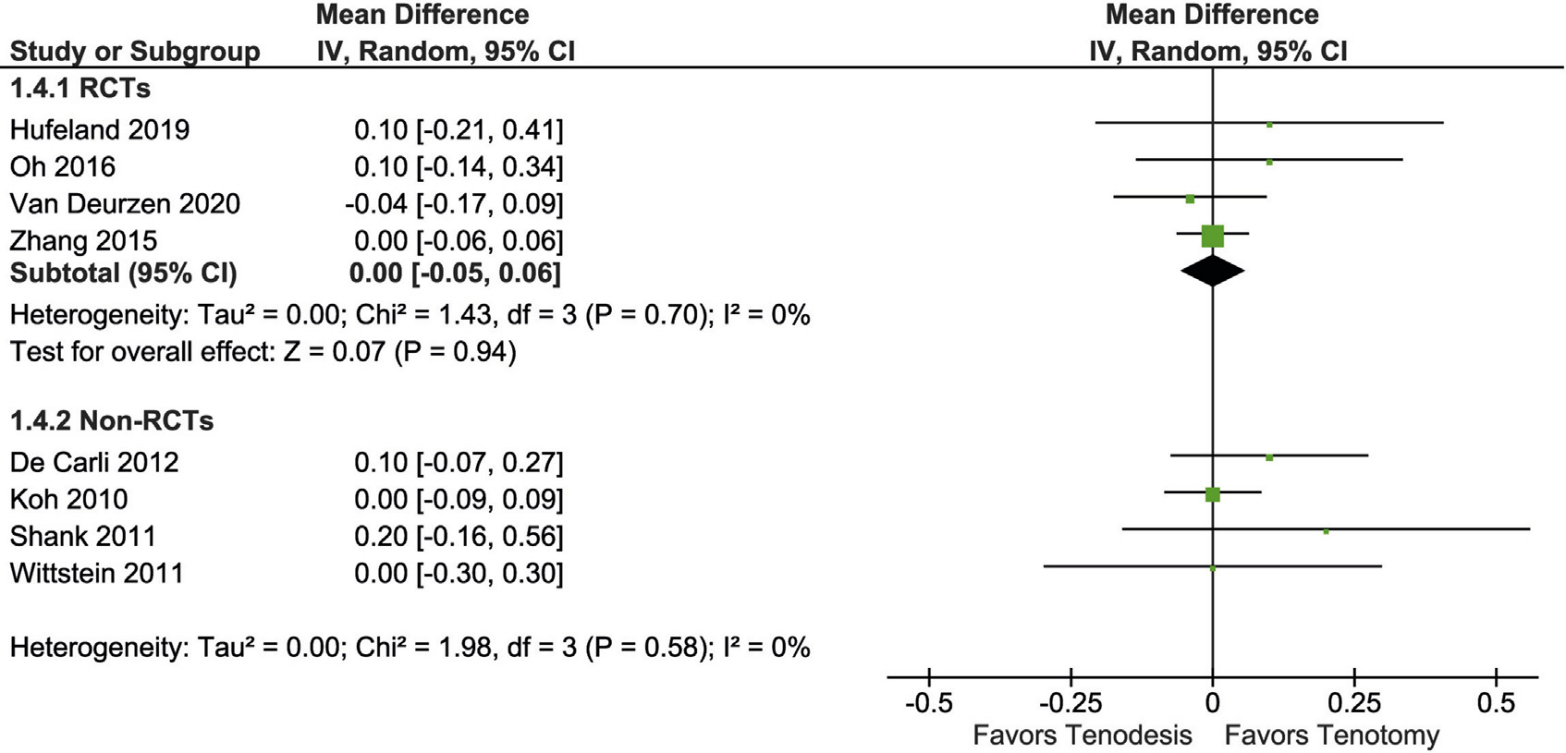
Forest plot of elbow flexion strength.

ESI was reported in 8 studies including 535 patients (Fig 6). In a meta-analysis of 4 RCTs (315 patients), the ESI was similar in both groups (MD, 0 loss of strength compared to the contralateral side, 95% CI, -5% to 6%; 96% PI, -12% to 12%).

### FSSI

**Fig 7:**
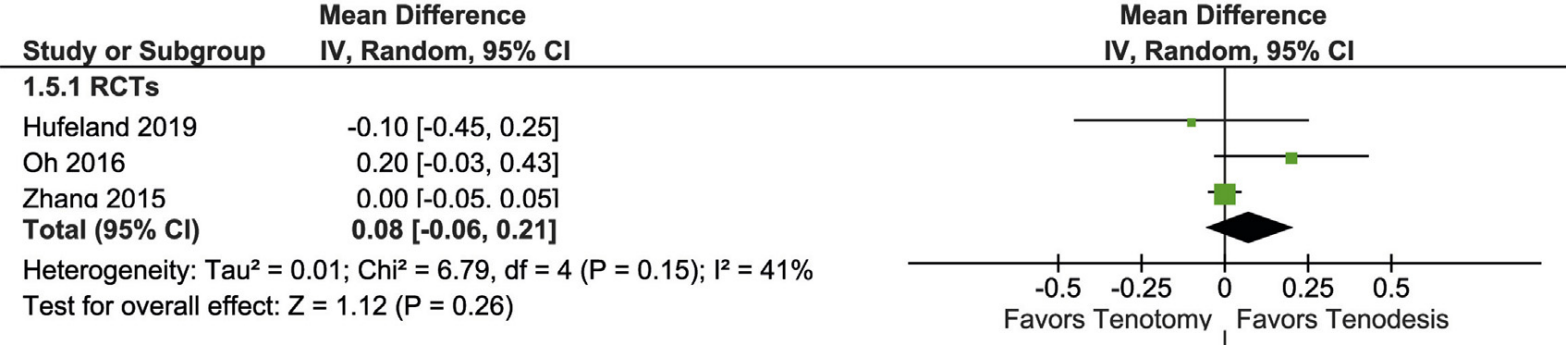
Forest plot of supination strength.

FSSI was reported in 5 studies including 300 patients (Fig 7). In a meta-analysis of 3 RCTs (329 patients), the FFSI was similar in both groups (MD, 3% loss of strength compared to the contralateral side in favor of tenodesis (95% CI, -10% to 16%; 96% PI, -123% to 129%).

### Cramping Bicipital Pain

**Fig 8:**
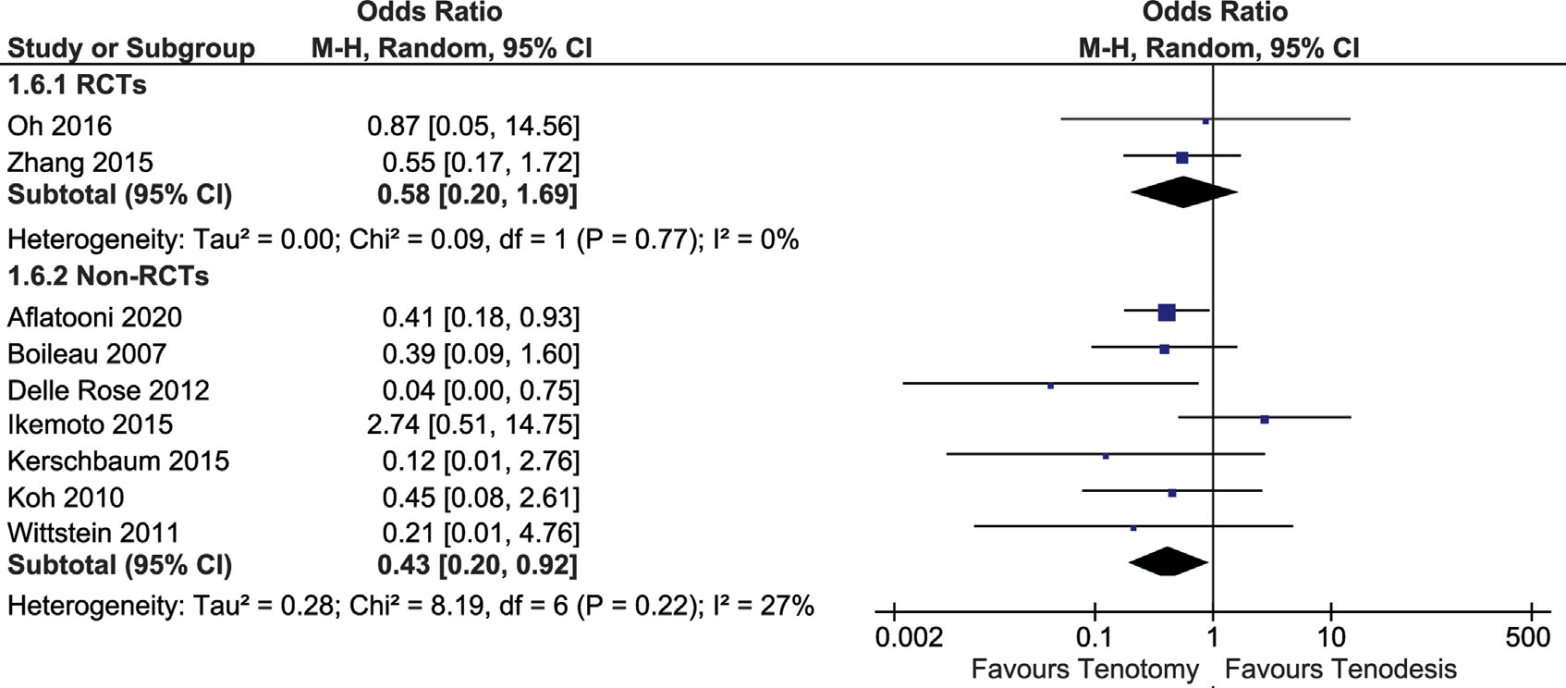
Forest plot of the presence of cramping pain.

Cramping pain in the biceps muscle was reported in 10 studies including 888 patients (Fig 8). These studies included 2 RCTs. In a meta-analysis of 2 RCTs (209 patients), there was no difference between the groups regarding cramping pain (OR, 0.58; 95% CI, 0.20-1.69)). In the nonrandomized studies, the OR for the presence of cramping pain ranged from 0.03 to 2.74 (I^2^ = 32%). Including only studies of patients with a mean age older than 60 years decreased heterogeneity substantially (I^2^ = 0%). Similarly, including only studies with <10% of concurrent cuff repairs and <10% of cointerventions decreased heterogeneity substantially (I^2^ = 2%).

## Discussion

The present meta-analysis did not demonstrate a clinically significant advantage of LHB tenodesis over tenotomy in terms of shoulder function, shoulder pain or biceps-related strength. Popeye deformity and cramping pain were more commonly observed in patients after tenotomy. Overall, the nature of the findings is similar to that of previous meta-analyses that also included nonrandomized studies in the pooling of data.^5^^,^^42^^,^^43^ However, the evidence base of our findings is much greater because we included only RCTs in the meta-analysis. Compared to the previous review,^5^ there were 16 new studies and 1,541 new patients; these included 7 new RCTs^15^^,^^20^^,^^26^^,^^30^^,^^33^^,^^39^^,^^40^ with a total of 511 new patients.

When strictly adhering to these findings, the only reason to perform an LHB tenodesis would be to reduce the likelihood of having a Popeye deformity or cramping bicipital pain. Indeed, a majority of patients preferred LHB tenodesis over tenotomy in a recent study, irrespective of their ages.^44^ The main reason for this preference was concern about upper-arm appearance. Yet in another recent study of 41 patients after LHB suprapectoral tenotomy (mean age 58 years, range 27-76), none of the 15 patients who developed a Popeye deformity had cosmetic complaints.^45^ In the same study, 26 patients developed autotenodesis of the LHB tendon stump in the intertubercular groove, as confirmed by ultrasound.^45^ These data may be used for counseling so patients are not concerned about the occurrence of a Popeye deformity.^46^ In our analysis, all LHB tenodesis techniques were analyzed together, and we did not find any clinically significant differences (that is, a difference equal to or larger than the minimal clinically important differences compared to LHB tenotomy. Yet a recent network meta-analysis by Anil and colleagues separately compared all tenodesis techniques (arthroscopic intracuff tenodesis, arthroscopic suprapectoral tenodesis and open subpectoral tenodesis) with tenotomy across 22 studies.^47^ It was concluded that all tenodesis techniques yield superior functional outcomes to those of tenotomy. The clinical significance of the observed differences (<5 for ASES, <4 for the Constant score), however, can be questioned, and our conclusion would be more conservative (i.e., that there is no clinical difference between tenodesis and tenotomy). Importantly, Anil and colleagues did find a clearly higher rate of persistent bicipital groove pain after intra-cuff tenodesis compared to tenotomy (OR, 2.9), which can be a sound argument to refrain from this tenodesis technique. Suprapectoral and subpectoral tenodeses had similar clinical improvements as was also confirmed by a recent focused comparative meta-analysis.^48^

It is of special interest in the present review that including or excluding lower-quality studies (that is, level 2 and 3 studies) did not change the results of the meta-analysis. Therefore, the usefulness of nonrandomized studies should not be underestimated.

Future randomized controlled trials concerning LHB tenotomy and tenodesis may stratify for patient age, may exclude patients with cointerventions and should incorporate patient-reported outcomes, including patient satisfaction. In this light, registry-based studies may offer sensible study designs to evaluate subgroups that may benefit from LHB tenodesis. Furthermore, based on our exploration of heterogeneity, future studies should include a more homogeneous age category (for example, only patients older than 60 years of age or only patients younger than 40 years of age) and should have a follow-up of at least 2 years.

### Limitations

Our meta-analysis has several limitations. First, the quality of the included studies is highly variable, as is evident from the wide range in Coleman scores. This limits the quality of the summary estimates of the meta-analysis. However, the results were not changed when analyzing only RCTs.

A second limitation is the high frequency of cointerventions in the included studies, mainly rotator cuff repair. Only 3 of 25 studies reported solely on patients with no concomitant procedures. Therefore, improvements in outcome parameters may be attributed to these cointerventions, so the isolated effect of LHB treatment can become hard to measure. Indeed, our subgroup analysis of studies with less than 10% of cointerventions resulted in very low heterogeneity for studies on bicipital pain.

Third, the outcome measures used in the studies may be insufficient.^49^ The Constant score may have a ceiling effect for LHB-related complaints in patients after rotator cuff repair^50^ because LHB tendinopathy causes mainly pain, not functional impairment. Elbow flexion and forearm supination strength were recorded only during a single measurement, not taking into account potential muscle fatigue. The LHB tendon may account for only 8%-20% of forearm supination strength,^20^ so these measurements may be insufficient to detect smaller differences that may be clinically important, mainly in younger patients. Using the LHB score may provide more specific information regarding LHB-related complaints.^51^ Ultimately, studies lack patient satisfaction measurements, which may be relatively important in a population where preoperative cosmetic concerns are prevalent.^44^

Fourth, no distinction is made regarding patient age or activity level. For example, younger and more active patients may benefit more from a tenodesis in terms of elbow flexion and/or forearm supination strength. One should be cautious about applying the present findings to all individuals.

The last limitation of the meta-analysis is that the location of the tenodesis is not analyzed separately. For example, it can be hypothesized that subpectoral tenodesis removes the LHB tendon entirely from the intertubercular groove, whereas higher tenodeses and the majority of tenotomies leave the tendon trapped in the groove. Some authors suggested that the surrounding tissues in the bicipital grove such as the transverse ligament may play a role in persisting pain after either LHB tenotomy or tenodesis.^52^^,^^53^ Persisting pain after either procedure may be explained in cases in which deroofing the bicipital groove has not been performed,^52^ which could clarify the similar results with regard to persisting pain in both groups in this meta-analysis. Surprisingly, Anil and colleagues found the lowest rates of persistent groove pain in groups that had undergone arthroscopic suprapectoral tenodesis,^47^ and a recent meta-analysis by van Deurzen and colleagues found no clinically relevant differences between suprapectoral and subpectoral tenodesis.

## Conclusions

In our meta-analysis, a Popeye deformity was more commonly observed in patients treated with tenotomy. Based on findings in a substantial number of studies, there is no evidence-based benefit of LHB tenodesis over tenotomy in terms of shoulder function, shoulder pain or biceps-related strength. It is unclear whether LHB tenodesis is of benefit in specific patient groups such as younger individuals.

## Supplementary Data

ICMJE author disclosure forms
